# Observations of extensive gene expression differences in the cerebellum and potential relevance to Alzheimer’s disease

**DOI:** 10.1186/s13104-018-3732-8

**Published:** 2018-09-04

**Authors:** Sally Chappell, Tulsi Patel, Tamar Guetta-Baranes, Fei Sang, Paul T. Francis, Kevin Morgan, Keeley J. Brookes

**Affiliations:** 1 Human Genetics, School of Life Sciences, University of Nottingham, Nottingham, UK; 2 DeepSeq Facility, University of Nottingham, Nottingham, UK; 3 Wolfson Centre for Age Related Diseases, King’s College London, London, UK

**Keywords:** RNA-sequence, Human brain, Cerebellum, Alzheimer’s disease

## Abstract

**Objectives:**

In order to determine how gene expression is altered in disease it is of fundamental importance that the global distribution of gene expression levels across the disease-free brain are understood and how differences between tissue types might inform tissue choice for investigation of altered expression in disease state. The aim of this pilot project was to use RNA-sequencing to investigate gene expression differences between five general areas of post-mortem human brain (frontal, temporal, occipital, parietal and cerebellum), and in particular changes in gene expression in the cerebellum compared to cortex regions for genes relevant to Alzheimer’s disease, as the cerebellum is largely preserved from disease pathology and could be an area of interest for neuroprotective pathways.

**Results:**

General gene expression profiles were found to be similar between cortical regions of the brain, however the cerebellum presented a distinct expression profile. Focused exploration of gene expression for genes associated with Alzheimer’s disease suggest that those involved in the immunity pathway show little expression in the brain. Furthermore some Alzheimer’s disease associated genes display significantly different expression in the cerebellum compared with other brain regions, which might indicate potential neuroprotective measures.

## Introduction

RNA-sequencing is now at the forefront of genetic research into complex disease, for accurate quantitation of gene expression and has been found to provide more comprehensive view of the RNA landscape than microarrays [1]. Whereas most expression studies have focused on differential gene expression between control and disease tissue, few investigate differential expression observed between different tissues from the same individual. In order to determine how gene expression is altered in disease or pre-disease state it is of fundamental importance that the global distribution of gene expression levels is understood and how this might differ between tissue types to inform accurate tissue choice for investigation of altered expression in disease state. Online databases such as the Genotype-Tissue Expression (GTEx) project [2] are extremely helpful in determining this, however there is a need to gain insight for gene expression profiles in tissues from the same samples, as individual differences between samples (e.g. genetic background) might mask true positive findings and extenuate false positive finding.

The Brains for Dementia Research (BDR) cohort is an initiative to provide tissue for investigation into the aetiology of dementia. The cohort consists of well-defined clinically and neuropathological post-mortem brain tissue from healthy control and dementia samples, with the majority diagnosed as Alzheimer’s disease (AD). Recent RNA-sequencing studies comparing AD and control tissue compare either a single region, or a few specific regions or cell-types of the brain, producing variable results for the genes displaying significant difference in expression between disease states [3–6]. This may be due to some genes showing expression differences between disease states in some regions but not others. Therefore in order to establish how reflective such studies are of each other, the gene expression profile across different brain regions must be conducted in corresponding tissue from the same subject. The discernment of which genes are differentially expressed between brain regions and not others could aid our understanding as to why certain regions of the brain are susceptible to various neurodegenerative diseases and help inform which region should be studied in relation to certain genes or pathways.

## Main text

### Methods

Three neuro-pathologically confirmed healthy controls were selected from the BDR repository. With three biological replicates per brain region, there is 87% power to detect gene expression fold-changes > 2 [7]. Tissue samples from five regions of the brain (frontal, temporal, occipital, parietal and cerebellum) were provided for each of the three samples. Two of the samples were female, with one male sample, the average age of death was 71 years (± 14 years), and average PMI = 50.3 h.

RNA was isolated from each of the regions using an in-house developed methodology preparing tissue with the Covaris cryoPrep system, to crush the tissue to increase the surface area and allowing efficient cell lysis in 1 ml TriZol following by RNA extraction with the RNAeasy Minikit from QIAGEN. A total of 2 μg of RNA was sent to the University of Nottingham DeepSeq Facility, for library preparation and sequencing. Total RNA samples were processed for ribosomal RNA depletion in order to enrich for non-coding and coding genes. Enriched RNA samples were then used to generate barcoded-sequencing libraries. Libraries were multiplexed onto 20 high output runs (2 × 75 bp) generating around 60 million reads per sample with sequencing performed using Illumina Nextseq500 platform.

The filtering pipeline was used to filter reads with low sequencing score as well as reads aligned to adaptor sequences. First, raw reads were trimmed against adaptors using ‘Sythe’ (https://github.com/vsbuffalo/scythe), then reads were quality trimmed using ‘Sickle’ (https://github.com/najoshi/sickle). Reads passing the filters were mapped onto the reference genome (hg19) in the context of known gene exon coordinates (Ensembl) using the ‘tophat’ mapping tool. (https://ccb.jhu.edu/software/tophat/index.shtml).

Read counts for each gene were calculated using ‘htseq-count’ (http://www-huber.embl.de/users/anders/HTSeq/doc/count.html). RPKM (reads per kilobase of transcript per million mapped reads) counts for all genes were calculated [8]. The RPKM is simply a normalized read count (stranded/sense reads) for a given gene. The read count of the exon-space of a gene is normalised against the total number of mapped reads against the total length of the gene’s exon-space. Genes with an average RPKM of < 1 were deemed non-discriminatory from background noise [9] and should be viewed with caution.

### Statistical analysis

The programme ‘DESeq2’ [10] was used to detect differentially expressed genes between brain regions in a pair-wise fashion. This analysis uses the gene counts, and corrects for dispersion using Bayes theorem. The final values for differential expression are adjusted with a Benjamini-Hochberg (false discovery rate—FDR) with a P value < 0.05 deemed as significant.

### Results

There was an average of 15,742 genes expressed across the 15 samples with an RPKM greater than 1, indicating gene expression above background noise [9]. Each region displayed a varying number of genes expressed, with the cerebellum expressing the greatest number of genes with an average of 16,576 across the three samples. The frontal, temporal, occipital and parietal cortex regions displayed expression of 15,450; 14,930; 15,995 and 15,757 genes, respectively. One-way ANOVA with post hoc Tukey revealed a significant difference between the number of genes expressed between the temporal and cerebellum regions (P = 0.023). No other comparisons were significant (P > 0.1). Across all regions the most highly expressed genes were *SRP* and *RNU* RNA genes, involved in translation and splicing mechanisms.

There was an average of 20,132 gene comparisons analysed per region (based on gene count), suggesting that around 1000 (5%) genes would be found to be differentially expression by chance at the alpha significance threshold (P < 0.05 FDR corrected). In differential analyses between cortex regions far fewer genes were identified than this. Comparisons between the cerebellum and each cortex region suggested that 6–9 times more than the expected number of genes by chance were observed to be significantly differentially expressed (Table 1).

**Table 1:**
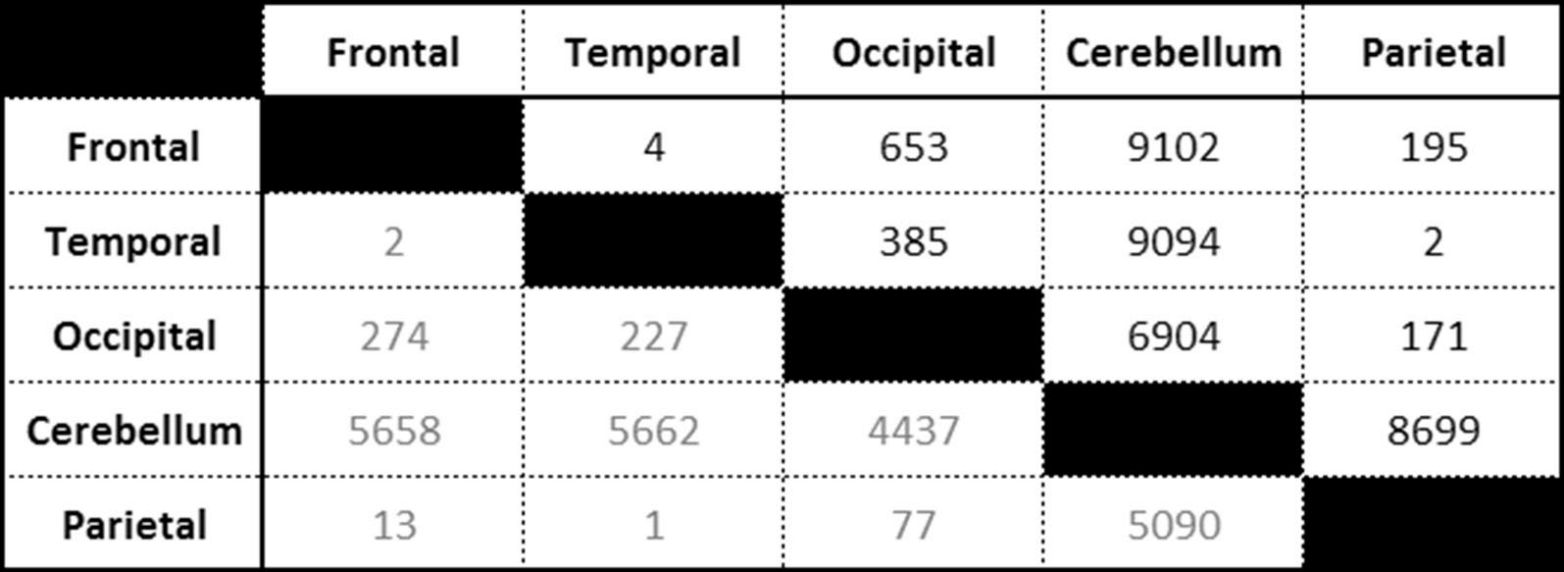
Numbers of significantly differentially expressed genes identified by ‘DESeq2’ between brain regions (Benjamini-Hochberg adjusted significance level of < 0.05; top right corner), and numbers of significantly differentially expressed genes filtered by fold-change of greater than doubled or halved expression changes (bottom left corner)

Gene expression in the cerebellum region was vastly different, with 11,770 unique genes differentially expressed compared to the other regions. Five thousand, three hundred and one genes (45%) were consistently differentially expressed between the cerebellum and the other cortex regions. The majority of these were concordant for direction of expression level change in the cerebellum compared to the other regions, with only 14 genes (0.3%) showing divergent expression direction changes between the cerebellum and each of the cortex regions.

Preliminary exploration of the data with Ingenuity’s Pathway Analysis (IPA) software (QIAGEN Bioinformatics) suggests that the common gene expression differences in the cerebellum indicate decreases in the development and quantity of neurons, and an increase in neuronal loss in the cerebellum. However it also suggests a decrease in long-term depression of the synapse and increases in long-term potentiation of cells, suggesting an increase in synaptic plasticity and therefore strength in the cerebellum.

**Fig. 1:**
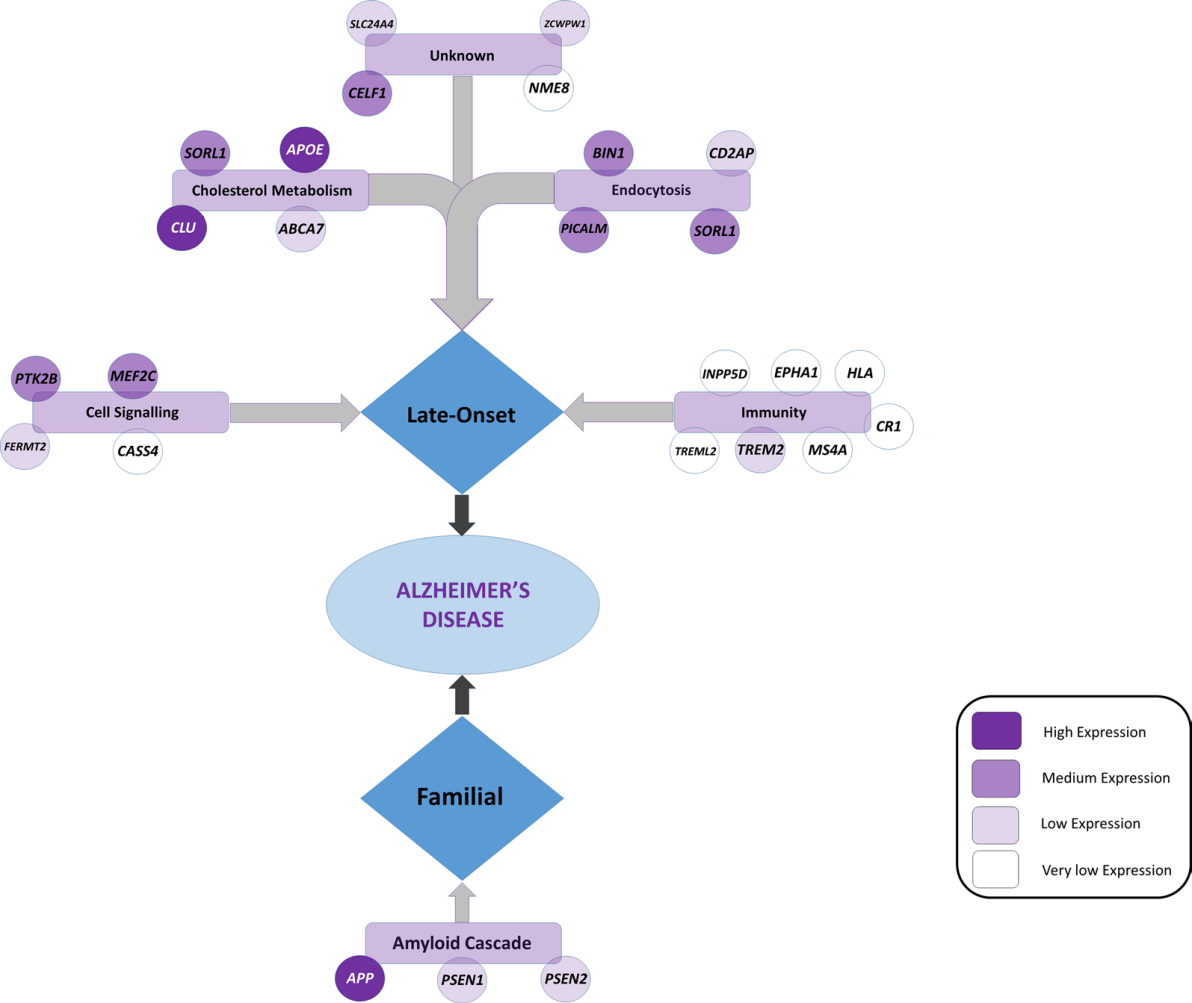
Graphic showing the mapping of key associated Alzheimer’s disease genes mapped on to pathways and their relative gene expression determined by RNA-sequencing RPKMs [8]. Genes involved in **Cholesterol Metabolism** and **Endocytosis** pathways are highly expressed in the brain, whilst genes involved in **Immunity** pathways show little expression in the brain (Figure Adapted from Medway and Morgan 2014)

Genes known to be involved in the familial early-onset form of AD, *APP, PSEN1*, and *PSEN2*, displayed varying levels of expression in the brain, with *APP* exhibiting high levels of expression, with *PSEN1* and *PSEN2* genes showing a lower level of expression (Fig. 1). Expression levels of *APP* and *PSEN1* were observed to significantly lower in the cerebellum, whereas *PSEN2* was higher.

The RNA-sequencing data for genes associated with the late-onset form of AD suggested considerable variation for the average level of gene expression (Fig. 1). Of particular note is that genes involved in immunity pathways were found to show very low levels of gene expression on average across all brain regions (RPKM ≤ 1), whilst those involved in cholesterol metabolism and endocytosis displayed moderate (RPKM > 15) to high gene (RPKM > 100) expression levels.

Nine of the late-onset form AD-associated genes displayed no significant changes in expression level between regions of the brain (*APOE, SORL1, TREM2, ABCA7, ZCWPW1, HLA*-*DRB5, MS4A64, CASS4,* and *TREML2*). Three genes displayed a significantly higher in expression in the cerebellum compared to all other cortex regions (*CELF1, CD2AP* and *EPHA1),* whilst six genes displayed a significantly lower expression in the cerebellum (*CLU, MEF2C, BIN1, FERMT2, SLC24A4* and *INPP5D)*. Finally four genes displayed no significant change in expression between the regions of the brain except in one comparison with the cerebellum (Table 2).

**Table 2:**
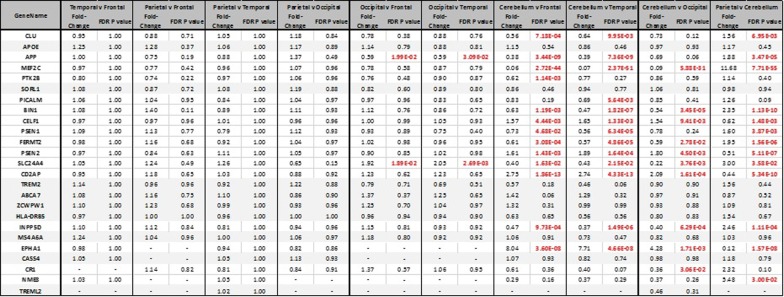
Table of DESeq2 results for Alzheimer’s disease related genes, indicating fold-change of expression in index brain region in comparison to the second brain region, and the Benjamini-Hochberg FDR corrected significance observed for the change. Those changes deemed significant with P < 0.05 are highlighted in red. Genes with no significant difference in expression level between any brain regions comparisons are shaded in grey

The data generated here has a high level of concordance with data from GTEx version 6 (v6). The comparison of medium RPKM values between our data and that obtained for brain regions available in GTEx are highly correlated for frontal (Pearson r = 0.974 P < 0.001) and cerebellum regions (Pearson r = 0.966 P < 0.001). Data from GTEx also supports the data for direction change in expression levels between the frontal and cerebellum regions, with 17 genes showing distinguishable levels of RPKM change between the regions, 14 of them (82.4%) are concordant with the data generated here. Discordant genes were *PTK2B, SORL1* and *BIN1*, showing the opposite direction of change in RPKM in the data provided here with that of GTEx v6.

### Discussion

The number of genes that exhibited expression differences between the different cortex regions of the brain were not above what was expected by chance, decreasing the credibility of those observations being true-positive findings. The key findings were the unique profile of the cerebellum gene expression, and the commonality of the genes differentially expressed between each of the four cortex comparisons. This echoes the findings by previous studies carried out using microarray technology [11–13]. These studies compared gene expression in the cerebellum to various other regions of the brain, and observed that while cortex regions had little variation of gene expression between them, when compared to the cerebellum over 1000 genes were found to be differentially expressed. The increased number of differentially expressed genes observed here, may reflect the greater accuracy and sensitivity of sequencing data over that of microarray data. Tentatively, the most interesting observation was from the pathway analysis which suggests that the cerebellum has increased synaptic plasticity and strength, with the gene expression changes suggesting an increase in long-term potentiation of the cells, despite a decrease in the development and quantity of neurons. Synapse plasticity and therefore strength of cell–cell signalling is thought to underlie learning and memory [14].

A previous investigation of gene expression changes related to aging across different regions of the brain identified that whereas several regions of the brain displayed similar age-related gene expression changes to the frontal cortex, the cerebellum displays little correlation with these regions [15]. Their analysis suggests that the cerebellum shows fewer gene expression changes in relation to aging compared to other parts of the brain, which may account for the large number and commonality of gene differentially expressed between the cerebellum and cortex regions presented here. One of the suggestions by Fraser et al. [15] was that the cerebellum could be aging at a slower rate than other regions of the brain, and these differentially expressed genes might represent those associated with aging.

The cerebellum has long thought to be relatively preserved in AD [16, 17], and PET studies have utilized the cerebellum as a pseudo-control investigating neuro-inflammation due to the lack of difference shown for TSPO density between patients with AD and controls [18, 19]. This might possibly suggest that the cerebellum has some protective measures against the onset of aging and/or AD pathology. This however requires further exploration and could be the basis of gaining insight into the preservation of neurons in the brain and therefore therapeutic intervention.

This was further supported by the observation of genes that are purportedly associated with AD (familial and sporadic) via association studies display higher or lower expression levels in the cerebellum compared to cortex regions.

It was found that some genes known to be involved in the familial and in the late-onset form of AD, were in fact expressed at low levels in the brain with some expressed at RPKM values below the cut-off that would indicate the levels could not be discriminated against background noise [9]. This was supported with expression data from GTEx v6. In particular genes known to be associated with late-onset AD, involved in the immunity pathway, now a leading focus for therapeutic investigation for the disease, are expressed at very low levels in the brain. Data from GTEx suggests these genes involved in the immunity pathway are highly expressed in tissues such as whole blood, the lung and spleen.

## Limitations

This pilot study lacks the power to discern real data on the variability between individuals. However it provides valuable data for gene expression across human brains regions for future reference. Ideally further RNA-sequencing of more specific brain regions on control and Alzheimer’s disease samples would add greater power to the study.

## Abbreviations

BDR: Brains for Dementia Research
RPKM: reads per kilobase of transcript per million mapped reads
FDR: false rate discovery
